# Nutrition practices and knowledge among NCAA Division III football players

**DOI:** 10.1186/s12970-017-0170-2

**Published:** 2017-05-19

**Authors:** Elizabeth Lea Abbey, Cynthia Joy Wright, Christina M. Kirkpatrick

**Affiliations:** Whitworth University, 300 W. Hawthorne Rd., Spokane, WA 99251 USA

**Keywords:** Nutrition source, Dietary recommendation, Football linemen, Registered dietitian nutritionist

## Abstract

**Background:**

Participation in collegiate American football is physically demanding and may have long-term health implications, particularly in relation to cardiovascular and neurological health. National Collegiate Athletic Association (NCAA) Division III (DIII) football players are a relatively unstudied population, particularly in terms of their dietary habits and knowledge. The aim of the present study was to descriptively evaluate the dietary intake of DIII football players including a subset of linemen and assess the nutritional knowledge and sources of information of these athletes.

**Methods:**

The study sample was 88 DIII football players including a subset of nine linemen. All participants completed a food frequency questionnaire, and a nutritional knowledge questionnaire that included a quiz and questions about their main sources of nutrition information. Heights and body masses were also recorded. The linemen submitted written 3-day diet records for assessment of their dietary intake.

**Results:**

Of the 88 participants, >50% reported consuming starches/grains, meat and dairy daily, but <50% reported consuming fruits and vegetables daily. Protein powders were the most commonly used supplements (33% reported daily use). Compared to dietary recommendations, linemen consumed high amounts of total fat, saturated fat, dietary cholesterol, sodium, and potassium, but were low in carbohydrates, fiber, and essential fats. The mean nutrition knowledge quiz score for the 88 participants was 55.2%. Those who had taken a nutrition or health course in college scored significantly higher on the quiz than those who had not. Participants reported relying primarily on coaches, websites, and athletic trainers (ATs) for nutritional guidance; ATs were the most trusted source.

**Conclusions:**

DIII football players had dietary habits that may both mitigate and increase their risk of chronic diseases. These athletes have room to improve their nutrition knowledge. Their reliance on athletic team staff for nutrition guidance highlights the importance of nutrition education for both athletes and staff and the potential role of a registered dietitian nutritionist.

**Electronic supplementary material:**

The online version of this article (doi:10.1186/s12970-017-0170-2) contains supplementary material, which is available to authorized users.

## Background

The long-term health of collegiate American football players, especially in relation to cardiovascular and neurological health, has received increasing attention from athletes, their families, coaches, and administrators. Previous studies involving former football players have shown that they have an increased risk of developing cardiovascular disease (CVD), neurodegenerative diseases, and a decreased health-related quality of life [1–4]. Since 1950, football players (particularly linemen) have had the largest increases in height, weight, and body mass index (BMI) for any collegiate sport [5]. Regardless of the level of collegiate competition, football linemen have higher body masses, waist circumferences, and BMIs than other position players [6–9]. Over time, increases in body composition measures may put linemen at a greater risk of developing metabolic syndrome, which can then lead to other diseases such as CVD and type two diabetes [6, 9–11].

While physical training plays a major role in alterations to body composition, the dietary practices of collegiate football players are a key component as well. There has been some research on the dietary intake and nutritional knowledge of NCAA Division I (DI) and Division II (DII) football players [8, 11–14] but little published research specific to NCAA Division III (DIII) athletes besides attitudes about nutrition [6]. Not only does nutrition have an impact on the cardiometabolic health of these athletes, but there is emerging evidence that it may be beneficial for neurological health as well [15–18]. This is of increasing concern in contact sports such as football.

Compared to DI and DII schools, DIII athletic programs tend to have limited nutrition-related resources available to their athletes (e.g. registered dietitian nutritionists) for both their acute and long-term needs. Even amongst larger collegiate programs, athletes tend to consult those with whom they have the most frequent contact (e.g. coaches, athletic trainers, and strength and conditioning specialists) [19]. Very few DIII football players will continue to play competitively past their collegiate careers and could potentially benefit from nutrition-related programming to help them transition out of competitive athletics. Before specific recommendations can be made, it is important to gain a background understanding of the nutritional practices and knowledge of this specific population. The objectives of this study were to 1) descriptively evaluate the dietary intake of NCAA DIII football players including a subset of the players at higher risk for cardiometabolic diseases (linemen), and 2) assess the nutritional knowledge and sources of information of these athletes.

## Methods

### Participants

Following approval of this study by the institutional review board of Whitworth University, a convenient sample of 88 NCAA DIII football players were recruited at either an informational session for returners held during the spring 2014 football season or via an e-mail sent to all new players prior to arrival at training camp in August of 2014. All participants provided written consent prior to their participation in the study. Due to resource and scheduling constraints, diet records could not be collected for all 88 study participants. Thus, due to their increased cardiometabolic disease risk, only linemen (*n* = 21) were invited to submit diet records for assessment of dietary intake, and 43% (*n* = 9) chose to participate in this additional data collection.

### Procedures

The study design was cross-sectional. Data was collected during sport physicals at the beginning of fall training camp. Athletic training students measured participant height using a measuring tape mounted on a wall to ±0.5 inches and converted to centimeters (cm). Weight was measured on a bathroom scale to ±0.5 lbs and converted to kilograms (kg). The participants then completed three questionnaires: a health history screening form, a food frequency questionnaire, and a nutrition knowledge questionnaire [19]. The food frequency questionnaire was developed for this study. Participants indicated the frequency with which they consumed 66 different foods and beverages, as well as four categories of supplements (i.e. protein powder, multivitamin/mineral, creatine, and other). The nutrition knowledge questionnaire was a 17-question, multiple-choice nutrition knowledge quiz based on one developed by Torres-McGehee et al. [19]. Question topics were related to macro- and micronutrients, supplements, weight management, and hydration [see Additional file 1]. The nutrition knowledge questionnaire also included questions on the participants’ top three sources of nutrition information, their comfort level with those sources, and the perceived adequacy of those sources. Finally, all participants reported whether they had taken a college nutrition and/or health course.

The linemen who agreed to submit three-day diet records met with a registered dietitian nutritionist the following morning to review diet record procedures and portion size estimations. Participants completed the records on consecutive days during training camp and were encouraged to consume their typical diets, recording everything that they ate and drank, including supplements.

### Data analysis

Food records were analyzed using Food Processor software (ESHA Research, Salem, OR). When a specific brand was not specified or available, the USDA Standard Reference was used. Dietary intake was assessed for energy, macronutrient, and micronutrient (sodium and potassium) content. These data were compared to the U.S. Dietary Reference Intake (DRI) standards and MyPlate recommendations. DRI and MyPlate recommendations were calculated using the mean height, body mass, age, and activity level (very active) of the linemen. The exceptions to this were the DRIs for carbohydrate and protein, which are widely recognized, athlete-specific recommendations. Recommended intakes of carbohydrate and protein were based on the guidelines outlined in the American College of Sports Medicine, Academy of Nutrition and Dietetics, and Dietitians of Canada Joint Position Statement on Nutrition and Athletic Performance [20]. Athletes who engage in moderate-to-high intensity exercise 1 – 3 h/day are advised to consume between 6 – 10 g/kg of carbohydrate per day. We set the recommended intake for the average linemen in our study at the mean of this range (8 g/kg/day). The amount of dietary protein believed to be necessary to support metabolic adaptations, repair, remodeling, and protein turnover is between 1.2 – 2.0 g/kg/day, spread throughout the day. The DRI for protein for the average lineman was set at the mean of this range (1.6 g/kg/day). MyPlate was released in 2011 by the U.S. Department of Agriculture (USDA) to replace the Food Pyramid. MyPlate recommendations use English measurements and are reported as such.

Statistical analyses were performed using SPSS v.20 (IBM, Armonk, NY). One-sample *t-*tests were used to compare nutrient levels to DRI standards and MyPlate recommendations. Differences in nutritional knowledge were investigated using independent *t*-tests comparing (a) individuals who reported taking a college nutrition course vs. those not taking a nutrition course, and (b) individuals who reported taking a college health course vs. those not taking a health course.

## Results

### Anthropometrics of the entire sample

Participant anthropometrics and demographics are reported in Table 1. Using the World Health Organization guidelines for body composition [21], the mean BMI for all subjects fell within the “overweight” category (BMI of 25 – 29.9 kg/m^2^) and the linemen subgroup within the “obese” category (BMI ≥30 kg/m^2^).

**Table 1:** Subject characteristics

Characteristic	Entire Sample (*n* = 88)	Linemen Subgroup (*n* = 9)
Average Age, yrs	19.6 ± 1.7	20.4 ± 1.5
Height, cm	180.6 ± 6.5	182.8 ± 6.3
Body Mass, kg	92.4 ± 16.1	113.9 ± 10.2
BMI, kg/m^2^	28.3 ± 4.2	34.2 ± 4.3
Years in College	2.1 ± 1.3	2.7 ± 1.4

Average intake numbers are mean ± standard deviation

### Nutrition practices in the linemen subgroup

**Fig. 1:**
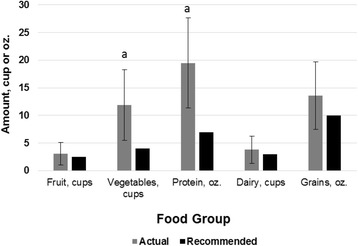
Actual intakes vs. MyPlate recommendations for linemen (*n* = 9). **a**, Significantly different than recommendations (*p* < .05)

Average energy, macronutrient, and micronutrient intakes of the linemen compared to the DRIs are reported in Table 2 and compared to MyPlate guidelines in Fig. 1. When compared to the DRIs calculated for the average lineman, participants were low in total carbohydrate, dietary fiber, and total polyunsaturated fats (PUFAs), including omega-3s and omega-6s. They had high intakes of total fat, saturated fat, dietary cholesterol, sodium, and potassium. The participants met the requirements for energy, monounsaturated fatty acids (MUFAs), and protein for an athlete. Their average protein consumption was 2.0 g/kg/d. The MyPlate daily guidelines for fruits, vegetables, protein, dairy, and grains for the average lineman were 2.5 c, 4.0 c, 7 oz, 3 c, and 10 oz. Participants met the requirements for fruits (3.1 c; t = 0.9, df = 8, *p* = 0.375), dairy (3.8 c; t = 1.0, df = 8, *p* = 0.347), and grains (13.6 oz; t = 1.8, df = 8, *p* = 0.111). They consumed high amounts of vegetables (11.9 c; t = 3.7, df = 8, *p* = 0.006) and protein (19.5 oz; t = 4.6, df = 8, *p* = 0.002).

**Table 2:** Average nutrient intake of linemen compared to the DRI

Nutrient	Dietary Intake (M ± SD)	DRI for Average Lineman^a^	*P* Value
Energy, kcals	5225.4 ± 1693.6	4552.9	.268
Total carbohydrate, g^a,b^	649.2 ± 261.5	911.2	.017^d^
Dietary fiber, g	45.8 ± 18.5	63.7	.020^d^
Protein, g^c^	225.0 ± 89.6	182.2	.190
Total fat, g	192.5 ± 60.2	141.7	.035^d^
Saturated fat, g	61.3 ± 17.3	45.5	.026^d^
MUFA, g	49.0 ± 15.7	50.6	.769
PUFA, g	29.2 ± 9.3	45.5	.001^d^
Omega-3 s, g	2.4 ± 0.7	4.6	<.001^d^
Omega-6 s, g	25.5 ± 8.7	40.5	.001^d^
Dietary cholesterol, mg	957.6 ± 406.3	300.0	.001^d^
Sodium, mg	9404.3 ± 3390.5	2300.0	<.001^d^
Potassium, mg	6298.1 ± 1986.5	4700.0	.042^d^

*DRI* Dietary Reference Intake, *MUFA* monounsaturated fatty acids, *PUFA* polyunsaturated fatty acids

^a^DRI for average lineman was calculated for a standardized individual with the average body mass, height, age and activity level of the linemen subgroup

^b^DRI for carbohydrate based off of 8 g/kg

^c^DRI for protein based off 1.6 g/kg

^d^Significant difference

### Nutrition practices of the entire sample

Food frequency questionnaire results for the entire sample are reported in Table 3. Participants reported eating an average of 3.4 ± 0.7 meals per day and dining out 2.5 ± 1.8 times per week. Of the restaurants the participants reported eating at regularly, 71% were either fast food or fast casual restaurants.

**Table 3:** Food frequency questionnaire data: % of participants (n = 88) who self-reported foods/beverages/supplements at specified frequencies

	Daily (7 d/wk)	Frequently (3–6 d/wk)	Weekly (1–2 d/wk)	Monthly (1/mo)	Rarely (<1/mo)	Never
Foods						
Starches/grains	67.0	30.7	2.3	----	----	----
Meat	52.3	39.8	6.8	1.1	----	----
Seafood	6.0	28.6	22.6	25.0	15.5	2.4
Dairy	82.8	13.8	2.3	1.1	----	----
Fruits	47.1	26.4	20.7	1.1	4.6	----
Vegetables	38.4	22.1	24.4	4.7	10.5	----
Desserts/candy	20.2	27.0	28.1	19.1	4.5	1.1
Beverages						
Sports drinks	34.1	31.8	21.6	5.7	2.3	4.5
Juice	29.9	19.5	29.9	10.3	6.9	3.4
Coffee	3.4	6.8	14.8	8.0	22.7	44.3
Soda	2.3	6.8	17.0	12.5	29.5	31.8
Energy drinks	2.3	4.6	6.9	11.5	21.8	52.9
Supplements						
Protein powders	33.0	23.9	11.4	3.4	13.6	14.8
Multivitamin/mineral	18.2	10.2	8.0	3.4	29.5	30.7
Creatine	5.7	5.7	5.7	4.5	18.2	60.2
Other	6.9	6.9	2.3	1.1	14.9	67.8

### Nutrition knowledge & information sources for the entire sample

Due to the small sample size of the linemen subgroup, nutrition knowledge and information sources are reported for the entire sample only. The mean nutrition quiz score was 55.2 ± 16.3%. Only 11.5% of participants had taken a nutrition course in college, and their mean score was significantly higher than those who had not (71.2 vs. 53.6%; t = -7.38, df = 85, *p* < 0.001). Participants who had taken a health course in college (15.3%) had a significantly higher mean score than those who had not (68.5 vs. 53.2%; t = -3.39, df = 84, *p* < 0.001).

The most commonly missed questions (<50% answered correctly) related to acceptable macronutrient distribution ranges for athletes, micronutrient function and toxicity, safety and regulation of ergogenic aids, body composition assessment, and recommended strategies for increasing muscle mass. Over 75% of participants correctly answered questions related to pre-exercise fueling, potential performance benefits of creatine supplementation, post-exercise rehydration guidelines, and possible side effects of electrolyte loss.

**Fig. 2:**
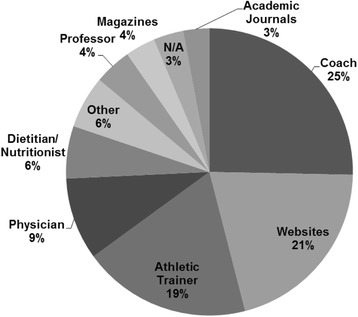
Sources of nutrition information (*n* = 88)

**Fig. 3:**
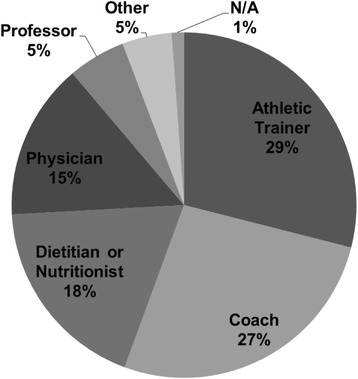
Person(s) with whom athletes were most comfortable discussing nutritional needs (*n* = 88)

**Fig. 4:**
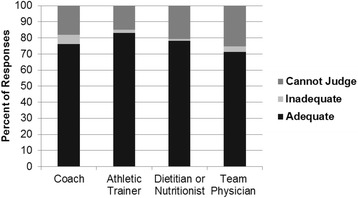
Perceived adequacy of nutrition source (*n* = 88)

Figures 2, 3 and 4 display the primary sources of nutrition information for participants, the person with whom they are most comfortable discussing nutrition topics, and the perceived adequacy of these sources. When assessing the perceived adequacy of the source (Fig. 4), participants selected “cannot judge” if they had not sought out nutrition information previously and/or did not clearly identify with the other descriptors (i.e. “adequate” and “inadequate”).

## Discussion

### Anthropometrics of the entire sample

Compared to NCAA DI and DII athletes, past researchers have reported that DIII players tend to have significantly lower body masses (DI = 131.3 kg, DII = 123.4 kg, DIII = 108.4 kg) [6]. The range of reported body masses for DIII football players varies from study to study, and our participants were on the low end of published data with a mean of 92.4 kg. Hoffman et al. [7] reported a range of 93.7 – 103.3 kg over five years, though Stuempfle et al. [22] reported a mean of 88.6 kg. However, when looking at research on body mass in DIII linemen specifically, our participants were on the higher end of mean body mass (113.9 kg for our linemen vs. 105.8 – 112.3 kg [7] and 96.6 – 101.0 kg [22]). The mean BMI for the entire sample was comparable to that reported by Stuempfle et al. [22] (28.3 kg/m^2^ vs. 27.4 kg/m^2^), however their offensive linemen had BMIs within the “overweight” category (29.9 kg/m^2^), while ours were considered obese (34.2 kg/m^2^). BMI has its limitations in athletic populations with muscular builds and should therefore be considered along with other body composition measurements such as waist circumference and percent body fat when assessing cardiometabolic disease risk. Regardless, the linemen in our study were well over the BMI cutoff to be classified as “obese” and would be at a higher cardiometabolic disease risk than their teammates in other positions.

### Nutrition practices in the linemen subgroup

Published studies on the dietary intakes of collegiate football players have been limited to DI athletes and have not been separated by position. To our knowledge, ours is the only assessment of the nutritional intake of DIII linemen specifically. Despite the small number of linemen in our study, the exploratory nature of this research and lack of information on this population in the literature warrant inclusion of the collected data.

The energy and macronutrient intakes of our participants were generally higher than those of other research groups [8, 13], though a direct comparison is difficult due to variables such as the time of testing (e.g. off season, pre-season, post-season), their playing positions, and year of eligibility (e.g. freshmen vs. all years). The DIII linemen in our study consumed excessive total fat, saturated fat, dietary cholesterol, sodium, and potassium, and had low intakes of carbohydrates, fiber, and PUFAs. Consumption of a diet high in saturated fat and sodium and low in fiber, PUFAs and MUFAs, has been associated with an increased risk of chronic diseases such as CVD [23, 24]. Conversely, consumption of fruits and vegetables, whole grains and foods high in PUFAs (e.g. omega-3 fats) and MUFAs have been shown to be cardioprotective [25–27]. Saturated fat has been primarily linked to CVD risk due to its potential to increase blood levels of LDL cholesterol, an effect that can be mediated by replacement with PUFAs and MUFAs [24]. PUFAs and MUFAs appear to have a cardioprotective effect through many potential mechanisms including decreased plasma triglycerides, resting heart rate, blood pressure, and inflammation, as well as improved vascular function and myocardial filling [23]. However, the current sample of linemen likely did not consume sufficient MUFAs or PUFAs to receive this protective effect. A couple of recommendations for this population would be to substitute oils for solid fats (e.g. olive oil vs. butter) and at least one serving a week of seafood for other animal products.

Dietary cholesterol intake for the linemen was over three times the previous recommended amount. It is important to note that the most recent Dietary Guidelines for Americans [28] no longer include the <300 mg/d recommendation since there is evidence that dietary cholesterol has less of an impact on blood cholesterol levels than other types of fats (e.g. saturated and *trans* fats). Dietary cholesterol is found only in animal products and is not an essential nutrient (i.e. it does not need to be consumed in the diet). The liver produces enough endogenous cholesterol to meet the body’s needs and will moderate production to balance exogenous cholesterol. The Dietary Guidelines emphasize that eating patterns that include lower intake of dietary cholesterol are associated with a lower risk of CVD, which is why we chose to include the previous DRI in our analysis. However, more research needs to be done to determine whether there is a benefit in isolating this particular aspect of the diet.

Athletes, particularly heavy sweaters, need more sodium than non-athletes [29], though sweat rates and sodium loss can vary widely among individuals. Godek et al. [30] observed an average sodium loss of 12.5 g over a 4.5-h practice in 14 NFL linemen. The range of sodium loss for players of all positions (*n* = 44) over that period was 2.5 – 30.2 g. The sodium intake of our subjects was over three times that of the recommendation for a healthy adult, but could be appropriate depending on their individual sweat rates. Guidelines for sodium intake in this active population should take into consideration each athlete’s unique physiology and needs. However, over the long-term, a diet high in sodium post-competitive athletics could result in adverse cardiovascular effects through a rise in blood pressure [31]. Both a dietary reduction in sodium and increase in potassium have been shown to help moderate blood pressure. Our participants met the MyPlate recommendations for fruits and exceeded them for vegetables, which likely helped them meet the DRI for potassium. Besides being high in potassium, diets rich in fruits and vegetables may be beneficial for cardiovascular health because of their high antioxidant content, which may minimize inflammation and oxidative stress [25, 32]. Fruits and vegetables, along with whole grains, are also excellent sources of dietary fiber. High-fiber diets appear to reduce inflammation and oxidative stress, improve blood lipid profiles and blood pressure, and regulate glucose metabolism. The linemen in our study did not meet the guidelines for fiber (72% of recommended; 45.8 vs. 63.7 g/d) despite meeting the MyPlate guidelines for grains and fruits and exceeding the guideline for vegetables. This may be because the grains group in MyPlate includes not only whole grains but refined flours and sugars as well.

When using the carbohydrate recommendation for an athlete who engages in moderate- to high-intensity exercise, our participants’ average intake (set as the median of the range from 6 – 10 g/kg body weight) was low (71% of recommended; 649.2 vs. 911.2 g/d). Depending on their individual levels of activity, this amount of carbohydrate intake may be adequate for most football players. For some athletes, however, a higher amount consumed throughout the day may be necessary to adequately replenish glycogen stores and provide supplementary fuel during exercise [33]. This is particularly important during periods of intense training, such as during training camp. Consuming carbohydrates throughout the day in the form of whole grains, fruits and vegetables, could also improve their dietary fiber intake and therefore their cardiometabolic risk.

The linemen had significantly lower intakes of total PUFAs and omega-3 fatty acids compared to the recommendations. Of the omega-3 fatty acids, docosahexaenoic acid (DHA) is the primary structural omega-3 in the brain [17]. In rodent studies, supplementation with DHA, either before or after concussions or mild traumatic brain injuries, has helped improve functional outcomes such as memory [17, 18, 34]. In the only known study on the impact of DHA supplementation on traumatic brain injury in humans, Oliver et al. [15] measured a biomarker of brain injury among DI football players who consumed varying doses of a DHA supplement (2 – 6 g/d) over the course of a competitive season. Irrespective of the dosage, the DHA-supplemented subjects had attenuated biomarkers of brain injury compared to the placebo group. Considering current worry about the long-term effects of football on brain health, DHA supplementation (on top of food) seems promising but needs further evidence, especially for dosing. In light of the importance of omega-3 s in brain health, the lowered consumption in our group is concerning, and future nutritional education geared towards this population should consider emphasizing the potential benefits of consuming foods high in omega-3s (e.g. fatty fish).

Our participants met the recommendation for protein intake for athletes, though they consumed almost three times the protein recommendation provided by MyPlate (19.5 vs. 7 oz.). Considering that other food groups on MyPlate are also significant sources of protein (e.g. dairy and grains), the overall protein intake of our participants appears to be very high when compared to the MyPlate recommendations for a “very active” individual. This calls into question the utility of using MyPlate for athletes since the guidelines do not appear to fully account for their unique nutritional needs during training. MyPlate may be a more helpful resource for general nutrition information outside of a training cycle and following an athlete’s competitive collegiate career.

### Nutrition practices of the entire sample

Over 80% of our participants reported consuming dairy products at least daily. The Dietary Guidelines for Americans 2015 – 2020 recommend that adults consume 3 c of low-fat or fat-free dairy products per day, particularly to ensure adequate intake of calcium and other nutrients essential for bone health [28]. Former football players who remain physically active may actually have a decreased risk of developing osteoporosis later in life compared to age- and BMI-matched controls [35]. Not only are dairy products an excellent source of calcium, they are commonly consumed as a recovery fuel, especially in beverage form, due to their mix of protein and carbohydrate, and their ability to aid in rehydration [36].

A majority of participants (67%) reported consuming starches/grains daily, though the amount that they ate is unknown. Considering that these foods are the primary sources of carbohydrate in the diet, some football athletes may not be consuming adequate amounts. This seems congruent with the low carbohydrate and fiber intake observed in the linemen in our study (see previous section).

The most widely consumed supplement amongst our participants was protein powder (68% of subjects reported at least weekly consumption). This is higher than that reported by Jonnalagadda et al. [11] where 16% of their participants reported taking protein shakes or amino acids at the time they were surveyed. In contrast, while the percentage of participants in our study had higher protein powder consumption, the percentage of participants reportedly consuming creatine was lower compared to Jonnalagadda et al. [11] (17 vs. 36%). In a study by Burns et al. [37], 40.3% of DI athletes (all sports) reported using a protein supplement and 31.4% reported taking creatine. In that study, the frequency of intake of the supplement (weekly or less often) was not clear. Considering that our participants consumed a relatively high proportion of animal products in comparison to fruits and vegetables, they may already be getting adequate amounts of dietary protein and could be compromising other aspects of their diets. Encouraging the adoption of a more balanced eating plan during college may be an appropriate avenue for disease prevention.

On a positive note, the amount of soda consumed by our participants was much lower than previous self-reported daily soda intake among college males (non-athletes) at a university in the southern United States (2 vs. 48%) [38]. Even considering potential regional differences, the daily consumption of soda amongst our participants was quite low even though they had unlimited access to it in the dining hall. If they are consuming sugar-sweetened beverages, the participants in our study at least seem to be selecting beverages that they perceive to have a health or performance benefit (i.e. juices and sports drinks). Besides contributing extra kcals and few nutrients, sugar-sweetened beverages are linked to increased risk of cardiometabolic diseases [39]. The low intake of soda amongst our participants aligns with declining trends in sugar-sweetened beverage consumption in the United States, which is at a 30-year low [40]. We were not able to assess our participants’ intake of other added sugars due to limitations in the dietary analysis software used. This is an important consideration for future research as the newly revised nutrition facts label will require identification of added sugars.

Another promising aspect of the participants’ dietary intake was that 57% of respondents reported consuming seafood weekly. According to National Health and Nutrition Examination Survey data on adult males, 84.2% consumed seafood in the past month [41]. As mentioned in the previous section on the dietary intake of the linemen, seafood, especially fatty fish and algal oils are excellent source of DHA and another omega-3 fatty acid (eicosapentaenoic acid). In addition to their role in potentially mitigating the effects of traumatic brain injury, researchers have been exploring how these nutrients may also be involved in the prevention of cognitive decline [42]. While it is not known whether higher seafood intake among football players is neuroprotective, the increasing evidence of cognitive impairment within this population warrants further investigation in this area [3, 4].

### Nutrition knowledge in the entire sample

The mean score of our participants (55.2%) was comparable to previous reports (54.9%) [19]. While there is no single nutrition knowledge quiz that has been used in this area of research, most include questions relating to macro- and micronutrient needs, supplement efficacy, and weight management. In a review of 29 studies assessing nutrition knowledge among athletes, common misconceptions included: the roles of nutrients and their energy content; protein acting as a primary energy source for muscles; vitamin and mineral supplements providing energy; and supplements being necessary to achieve peak performance [43]. We found that the majority of our participants were misinformed about similar topics. Some additional education on topics such as the functions of specific nutrients and the safety and regulation of supplements may prove beneficial. While we did not assess the relationship between nutrition knowledge and dietary habits, it is important to note that knowledge does not necessarily translate to behavior. In reviews on this topic, it appears that nutrition knowledge may have a slightly positive impact on healthy nutrition behaviors, though most correlations are weak [43, 44]. If nothing else, there does not appear to be a negative effect of increased nutrition knowledge. We did observe that our subjects who had taken a nutrition and/or health class in college performed better on the nutrition knowledge quiz compared to those who had not taken a class.

Finally, our participants reported that they relied primarily on coaches, websites and athletic trainers for nutritional guidance. Of the athletic team staff, the subjects appeared to put the most trust in athletic trainers to provide accurate nutrition information, which aligns with previous research [37]. Since most DIII schools do not employ a registered dietitian nutritionist, athletic departments should ensure that athletic trainers and coaches are equipped to provide basic nutritional advice and are educated on the limits within their scopes of practice.

### Limitations

To begin with, the purpose of this research was to descriptively evaluate the nutritional practices and knowledge of DIII football players, so conclusions on the factors that contributed to our observations (e.g. training or access to nutritional support) and the potential implications of these factors cannot be made. Second, all participants were from one university, which may not reflect the habits of DIII football players nationwide. Third, all data, except for anthropometric measurements, was self-reported by the participants. Since the participants had a limited amount of time to complete the questionnaires and were constrained by their tight schedules during training camp, more direct assessments (e.g. interviews and observations of food intake) were not feasible. The linemen were instructed by a registered dietitian nutritionist on how to estimate portion sizes, but there may have been inaccuracies in their reporting. Fourth, the subset of linemen studied was quite small at just nine participants, and there was a wide range in the amount of food consumed among participants. Even though they were instructed to maintain their typical eating patterns, they were in the midst of their fall training camp and were practicing more frequently than during the school semester. This, and the fact that they were eating a majority of their meals in a dining hall where they had unlimited access, may have contributed to higher daily food intake and a different composition of foods than they would eat during the rest of the season. Fifth, while the nutrition knowledge quiz used was based on a survey that had been determined to have construct validity, some changes were made to it to account for the specific population studied (e.g. removal of a question on the female athlete triad). There are also difficulties assessing what constitutes adequate nutrition knowledge and whether knowledge translates into significant behavior change. Finally, such quizzes assume that there is only one correct answer and that nutrition science and guidelines remain static. While no instrument is perfect, the chosen quiz was deemed the easiest and most practical way to assess nutrition knowledge in our sample.

## Conclusions

The long-term health of collegiate football players has received increasing attention, particularly as it relates to cardiometabolic and neurological health. The NCAA DIII football players in our study had dietary habits that may both mitigate and increase their chronic disease risk later in life. In addition, their overall sport nutrition knowledge was lacking and could potentially be improved by increased education. The athletes in our study relied primarily on coaches, the internet, and athletic trainers for this information, trusting that what they were receiving was accurate. Athletic staff therefore have a responsibility to understand the nutritional landscapes of their teams and encourage healthy eating habits that may have long-lasting effects. Increased educational opportunities for athletic staff may better equip them to provide basic nutritional advice and know when to refer athletes to a registered dietitian nutritionist.

## Additional file

Additional file 1: Nutrition Knowledge Questionnaire KEY.doc; Nutrition Knowledge Questionnaire KEY; Questionnaire includes questions on subject background information (e.g. nutrition/health courses taken), primary sources of nutrition information, perceived adequacy of nutrition sources, and a 17-question nutrition knowledge quiz. (DOCX 21 kb)

## References

[1] Simon JE, Docherty CL (2014). Current health-related quality of life is lower in former Division I collegiate athletes than in non-collegiate athletes. Am J Sports Med.

[2] Dobrosielski DA, Rosenbaum D, Wooster BM, Merrill M, Swanson J, Moore JB, Brubaker PH (2010). Assessment of cardiovascular risk in collegiate football players and nonathletes. J Am Coll Health.

[3] Lehman EJ, Hein MJ, Baron SL, Gersic CM (2012). Neurodegenerative causes of death among retired National Football League players. Neurology.

[4] Seichepine DR, Stamm JM, Daneshvar DH, Riley DO, Baugh CM, Gavett BE, Tripodis Y, Martin B, Chaisson C, McKee AC, Cantu RC, Nowinski CJ, Stern RA (2013). Profile of self-reported problems with executive functioning in college and professional football players. J Neurotrauma.

[5] Yamamoto JB, Yamamoto BE, Yamamoto PP, Yamamoto LG (2008). Epidemiology of college athlete sizes, 1950s to current. Res Sports Med.

[6] Buell JL, Calland D, Hanks F, Johnston B, Pester B, Sweeney R, Thorne R (2008). Presence of metabolic syndrome in football linemen. J Athl Train.

[7] Hoffman JR, Ratamess NA, Kang J (2011). Performance changes during a college playing career in NCAA division III football athletes. J Strength Cond Res.

[8] Kirwan RD, Kordick LK, McFarland S, Lancaster D, Clark K, Miles MP (2012). Dietary, anthropometric, blood-lipid, and performance patterns of american college football players during 8 weeks of training. Int J Sport Nutr Exerc Metab.

[9] Mathews EM, Wagner DR (2008). Prevalence of overweight and obesity in collegiate american football players, by position. J Am Coll Health.

[10] Wilkerson GB, Bullard JT, Bartal DW (2010). Identification of cardiometabolic risk among collegiate football players. J Athl Train.

[11] Jonnalagadda SS, Rosenbloom CA, Skinner R (2001). Dietary practices, attitudes, and physiological status of collegiate freshman football players. J Strength Cond Res.

[12] Rosenbloom CA, Jonnalagadda SS, Skinner R (2002). Nutrition knowledge of collegiate athletes in a Division I National Collegiate Athletic Association institution. J Am Diet Assoc.

[13] Cole CR, Salvaterra GF, Davis JEJ, Borja ME, Powell LM, Dubbs EC, Bordi PL (2005). Evaluation of dietary practices of National Collegiate Athletic Association Division I football players. J Strength Cond Res.

[14] Judge LW, Kumley RF, Bellar D, Pike KL, Pierson EE, Weidner T, Pearson D, Friesen CA. Hydration and Fluid Replacement Knowledge, Attitudes, Barriers, and Behaviors of NCAA Division 1 American Football Players. J Strength Cond Res. 2016, 2016. 10.1519/JSC.0000000000001397 26950346

[15] Oliver JM, Jones MT, Kirk KM, Gable DA, Repshas JT, Johnson TA, Andréasson U, Norgren N, Blennow K, Zetterberg H (2016). Effect of docosahexaenoic acid on a biomarker of head trauma in american football. Med Sci Sports Exerc.

[16] Schober ME, Requena DF, Abdullah OM, Casper TC, Beachy J, Malleske D, Pauly JR (2016). Dietary docosahexaenoic acid improves cognitive function, tissue sparing, and magnetic resonance imaging indices of edema and white matter injury in the immature rat after traumatic brain injury. J Neurotrauma.

[17] Barrett EC, McBurney MI, Ciappio ED (2014). ω-3 fatty acid supplementation as a potential therapeutic aid for the recovery from mild traumatic brain injury/concussion. Adv Nutr.

[18] Mills JD, Hadley K, Bailes JE (2011). Dietary supplementation with the omega-3 fatty acid docosahexaenoic acid in traumatic brain injury. Neurosurgery.

[19] Torres-McGehee TM, Pritchett KL, Zippel D, Minton DM, Cellamare A, Sibilia M (2012). Sports nutrition knowledge among collegiate athletes, coaches, athletic trainers, and strength and conditioning specialists. J Athl Train.

[20] Thomas DT, Erdman KA, Burke LM (2016). American college of sports medicine joint position statement. nutrition and athletic performance. Med Sci Sports Exerc.

[21] WHO. Obesity and overweight fact sheet. [http://www.who.int/mediacentre/factsheets/fs311/en/].

[22] Stuempfle KJ, Katch FI, Petrie DF (2003). Body composition relates poorly to performance tests in NCAA Division III football players. J Strength Cond Res.

[23] Michas G, Micha R, Zampelas A (2014). Dietary fats and cardiovascular disease: putting together the pieces of a complicated puzzle. Atherosclerosis.

[24] Siri-Tarino PW, Sun Q, Hu FB, Krauss RM (2010). Saturated fat, carbohydrate, and cardiovascular disease. Am J Clin Nutr.

[25] Widmer RJ, Flammer AJ, Lerman LO, Lerman A (2015). The Mediterranean diet, its components, and cardiovascular disease. Am J Med.

[26] Wang X, Ouyang Y, Liu J, Zhu M, Zhao G, Bao W, Hu FB (2014). Fruit and vegetable consumption and mortality from all causes, cardiovascular disease, and cancer: systematic review and dose-response meta-analysis of prospective cohort studies. BMJ.

[27] Cho SS, Qi L, Fahey GCJ, Klurfeld DM (2013). Consumption of cereal fiber, mixtures of whole grains and bran, and whole grains and risk reduction in type two diabetes, obesity, and cardiovascular disease. Am J Clin Nutr.

[28] Dietary Guidelines for Americans 2015–2020. 8th Edition. [https://health.gov/dietaryguidelines/2015/guidelines/].

[29] Casa DJ, Armstrong LE, Hillman SK, Montain SJ, Reiff RV, Rich BS, Roberts WO, Stone JA (2000). National athletic trainers’ association position statement: fluid replacement for athletes. J Athl Train.

[30] Godek SF, Peduzzi C, Burkholder R, Condon S, Dorshimer G, Bartolozzi AR (2010). Sweat rates, sweat sodium concentrations, and sodium losses in 3 groups of professional football players. J Athl Train.

[31] Gay HC, Rao SG, Vaccarino V, Ali MK (2016). Effects of different dietary interventions on blood pressure: systematic review and meta-analysis of randomized controlled trials. Hypertension.

[32] Liu R (2013). Health-promoting components of fruits and vegetables in the diet. Adv Nutr.

[33] Burke LM, Hawley JA, Wong SHS, Jeukendrup AE (2011). Carbohydrates for training and competition. J Sport Sci.

[34] Wu A, Ying Z, Gomez-Pinilla F (2014). Dietary strategy to repair plasma membrane after brain trauma: implications for plasticity and cognition. Neurorehabil Neural Repair.

[35] Lynch NA, Ryan AS, Evans J, Katzel LI, Goldberg AP (2007). Older elite football players have reduced cardiac and osteoporosis risk factors. Med Sci Sports Exerc.

[36] James L (2012). Milk protein and the restoration of fluid balance after exercise. Med Sport Sci.

[37] Burns RD, Schiller MR, Merrick MA, Wolf KN (2004). Intercollegiate student athlete use of nutritional supplements and the role of athletic trainers and dietitians in nutrition counseling. J Am Diet Assoc.

[38] West DS, Bursac Z, Quimby D, Prewitt TE, Spatz T, Nash C, Mays G, Eddings K (2006). Self-reported sugar-sweetened beverage intake among college students. Obesity.

[39] Malik VS, Hu FB (2015). Fructose and cardiometabolic health: what the evidence from sugar-sweetened beverages tells us. J Am Coll Cardiol.

[40] Kell J. Soda consumption falls to a 30-year low in the US. Fortune. 2016.

[41] Jahns L, Raatz SK, Johnson LK, Kranz S, Silverstein JT, Picklo MJ (2014). Intake of seafood in the US varies by age, income, and education level but not by race-ethnicity. Nutrients.

[42] Kesse-Guyot E, Péneau S, Ferry M, Jeandel C, Hercberg S, Galan P (2011). Thirteen-year prospective study between fish consumption, long-chain n-3 fatty acids intakes and cognitive function. J Nutr Health Aging.

[43] Heaney S, O'Connor H, Michael S, Gifford J, Naughton G (2011). Nutrition knowledge in athletes: a systematic review. Int J Sport Nutr Exerc Metab.

[44] Spronk I, Kullen C, Burdon C, O’Connor H (2014). Relationship between nutrition knowledge and dietary intake. Br J Nutr.

