# Supplementary material for: Nutrition practices and knowledge among NCAA Division III football players
Source: J Int Soc Sports Nutr. 2017 May 19;14:13. doi: 10.1186/s12970-017-0170-2 (PMC5437483; doi:10.1186/s12970-017-0170-2)
Supplement: Additional file 1: — Nutrition Knowledge Questionnaire KEY.doc; Nutrition Knowledge Questionnaire KEY; Questionnaire includes questions on subject background information (e.g. nutrition/health courses taken), primary sources of nutrition information, perceived adequacy of nutrition sources, and a 17-question nutrition knowledge quiz. (DOCX 21 kb) [file 12970_2017_170_MOESM1_ESM.docx]

**Nutrition Knowledge Questionnaire**

*Background Information*

How many years of college have you completed? _________________

What is your major? _________________________________

Have you taken a nutrition course in college? _______________ If so, when? __________________

Have you taken a health class in college? _______________ If so, when? __________________

*Nutrition Sources*

1. Who/what are your primary source(s) for nutrition information? Rank your top three.

_________Websites

_________Popular magazines

_________Academic journals

_________Coach

_________Athletic Trainer

_________Dietitian or Nutritionist

_________Physician

_________Professor

_________Other _______________________

_________Not Applicable—I have never sought nutrition information.

1. Who do you feel most comfortable discussing your nutritional needs with? Rank your top three.

_________Coach

_________Athletic Trainer

_________Dietitian or Nutritionist

_________Physician

_________Professor

_________Other _______________________

_________Not Applicable—I have never sought nutrition information.

1. In your experience, how would you rate the adequacy of the nutritional knowledge of the following people?

Coach Adequate Inadequate Cannot Judge

Athletic Trainer Adequate Inadequate Cannot Judge

Dietitian or Nutritionist Adequate Inadequate Cannot Judge

Team Physician Adequate Inadequate Cannot Judge

*Nutrition Knowledge: Answer the following questions to the best of your knowledge.*

1. An athlete’s diet should consist of approximately _________ protein _________ fat, and ________ carbohydrates.
   1. 12% to 15%, 25% to 30%, 55 to 70%
   2. 8% to 10%, 40% to 45%, 45% to 55%
   3. 25% to 35%, 55% to 65%, 70% to 90%
   4. 40% to 50%, 10% to 20%, 30% to 40%
2. A megadose of which of the following vitamins is potentially very dangerous?
   1. Thiamin
   2. Vitamin B6
   3. Vitamin C
   4. Vitamin A
3. From a sports performance perspective, which is the most significant and/or detrimental dietary deficiency?
   1. Iron
   2. Zinc
   3. Calcium
   4. Vitamin C
4. When dining at a fast food restaurant, a healthier low-fat food selection would be:
   1. Crispy chicken sandwich
   2. Green salad with ranch and cheese
   3. Grilled chicken sandwich
   4. Medium French fry
5. The optimal timing for consuming a post-exercise meal to restore glycogen (muscle carbohydrate stores) would be:
   1. Immediately after exercise
   2. Wait until you feel hungry
   3. 1 – 3 hours post-exercise
   4. 4 hours post-exercise
6. Which of the following is not a physiological effect of caffeine?
   1. Decreases the metabolic rate
   2. Stimulates the central nervous system
   3. Increases the secretion of epinephrine
   4. Increases heart rate and force of contraction
7. What is wrong with an athlete eating a 12- to 16-ounce Ribeye steak, baked potato with butter, green beans and soda 2 hours prior to an event?
   1. Sugar in the soda may take as long as 3 hours to metabolize
   2. The high-fat meal will take longer to digest and hinder performance
   3. Nothing; the pre-event meal should be at least 500 calories or more
   4. Nothing; the athlete should eat what makes him or her feel comfortable
8. Recent research has suggested that creatine supplementation may enhance performance in which of the following types of physical performance tasks?
   1. High intensity, anaerobic exercise such as powerlifting
   2. Cross Country competition event lasting about 30 minutes
   3. Marathon running (26.2 miles)
   4. Ultra marathons, such as Iron Man type triathlons
9. Which of the following statements regarding ergogenic aids (meant to improve sport performance) is false?
   1. Use of any aid that enhances sport performance is illegal and is grounds for disqualification.
   2. Although most nutritional ergogenic aids are safe, some dietary supplements pose significant health risks.
   3. Endorsement of a nutritional ergogenic aid by a professional athlete does not necessarily mean that it is effective as advertised.
   4. Some nutritional supplements marketed as ergogenic may contain prohibited drugs.
10. The recommended guidelines for safe and healthy weight loss are:
    1. 3 – 5 pounds per week
    2. 1 – 2 pounds per week
    3. 8 – 10 pounds per week
    4. 15 pounds a month
11. An athlete has been diagnosed with bulimia and has a known history of laxative abuse. Complications of chronic laxative use include which of the following?
    1. Electrolyte imbalance and dehydration
    2. Hyperactivity
    3. Vomiting blood
    4. Chronic nasal congestion
12. To safely increase muscle mass, it is recommended to increase both _____________ and ______________.
    1. Fat intake, carbohydrate intake
    2. Resistance training, caloric intake
    3. Resistance training, protein only
    4. Protein, water intake
13. All of the following are methods of measuring body composition (specifically body fat%) except:
    1. Body mass index (BMI)
    2. Underwater (“hydrostatic”) weighing
    3. Bioelectrical impedance analysis
    4. Measuring skin-fold thickness
14. During prolonged endurance exercise in the heat, excessive intake of water and inadequate intake of salt may lead to the life-threatening health condition:
    1. Hypertension (high blood pressure)
    2. Dehydration (fluid loss)
    3. Hyponatremia (water intoxication)
    4. Hyperkalemia (high potassium)
15. What measure is the best method to determine the amount of fluid loss due to sweat during an exercise session in which the athlete did not drink or go to the bathroom?
    1. Monitoring urine color
    2. Pre-post practice weigh-ins
    3. Thirst
    4. Urination frequency
16. If an athlete loses one pound of fluid during an exercise session, what recommended ounces of fluid should he/she drink after exercise?
    1. 16 – 24 fl. oz.
    2. 7 – 10 fl. oz.
    3. 24 – 36 fl. oz.
    4. 6 – 8 fl. oz.
17. Significant losses of electrolytes (such as sodium, chloride, potassium, or magnesium) during heavy exercise may lead to symptoms such as ____________ or ______________.
    1. Drop in blood pressure, increased production of urine
    2. Stress fracture, swelling
    3. Dyspnea (difficult or labored breathing), indigestion
    4. Muscular cramps, heat illness
